# Use of neuroimaging to measure neurocognitive engagement in health professions education: a scoping review

**DOI:** 10.1080/10872981.2021.2016357

**Published:** 2022-01-10

**Authors:** Serkan Toy, Dana D Huh, Joshua Materi, Julie Nanavati, Deborah A. Schwengel

**Affiliations:** a Department of Anesthesiology & Critical Care Medicine, The Johns Hopkins University School of Medicine, Baltimore, Maryland, USA; b The Johns Hopkins School of Medicine, Baltimore, Maryland, USA; c Welch Medical Library, The Johns Hopkins School of Medicine, Baltimore, Maryland, USA

**Keywords:** Electroencephalography, functional magnetic resonance imaging, functional near-infrared spectroscopy, health professions education, medical education, neurocognitive engagement, neuroimaging

## Abstract

**Purpose:**

To map the current literature on functional neuroimaging use in medical education research as a novel measurement modality for neurocognitive engagement, learning, and expertise development.

**Method:**

We searched PubMed, Embase, Cochrane, ERIC, and Web of Science, and hand-searched reference lists of relevant articles on April 4, 2019, and updated the search on July 7, 2020. Two authors screened the abstracts and then full-text articles for eligibility based on inclusion criteria. The data were then charted, synthesized, and analyzed descriptively.

**Results:**

Sixty-seven articles published between 2007 and 2020 were included in this scoping review. These studies used three main neuroimaging modalities: functional magnetic resonance imaging, functional near-infrared spectroscopy, and electroencephalography. Most of the publications (90%, n = 60) were from the last 10 years (2011–2020). Although these studies were conducted in 16 countries, 68.7% (n = 46) were from three countries: the USA (n = 21), UK (n = 15), and Canada (n = 10). These studies were mainly non-experimental (74.6%, n = 50). Most used neuroimaging techniques to examine psychomotor skill development (57%, n = 38), but several investigated neurocognitive correlates of clinical reasoning skills (22%, n = 15).

**Conclusion:**

This scoping review maps the available literature on functional neuroimaging use in medical education. Despite the heterogeneity in research questions, study designs, and outcome measures, we identified a few common themes. Included studies are encouraging of the potential for neuroimaging to complement commonly used measures in education research and may help validate/challenge established theoretical assumptions and provide insight into training methods. This review highlighted several areas for further research. The use of these emerging technologies appears ripe for developing precision education, establishing viable study protocols for realistic operational settings, examining team dynamics, and exploring applications for real-time monitoring/intervention during critical clinical tasks.

## Introduction

Learning in medicine is a complex process with many competing demands on trainees’ time and cognitive resources. The burden is further compounded by the high stakes that hinge on a trainee’s clinical performance. Research that could improve upon the current state of health professions education will require multimodal evidence to be impactful. The abundance of health professions education resources and their financial cost further necessitate scientific inquiry and validation of what exactly constitutes an appropriate medical education.

Historically, evidence based on self-report, multiple choice examinations, and behavioral observation dominated education research. More recently, neuroscience has made remarkable strides in helping us to understand neurocognitive processes [1]. Indeed, neuroimaging technology offers significant opportunities for health professions education research, but adoption of neurocognitive evidence in such research has lagged behind that for kindergarten-12 and higher education. Comprehensive reviews have been published to evaluate neuroimaging use with those learner groups [1]. In contrast, except for a few brief descriptive literature reviews limited in scope and specific to surgery [2–6], health professions education lacks such a robust review. Given that we were not able to find a comprehensive review of the general health professions education research on this emerging topic, we decided to use a scoping review methodology to explore the breadth and depth of this literature [7].

Our aim in conducting this review was to carefully map the literature on functional neuroimaging use in health professions education as an emerging, novel measurement modality for neurocognitive engagement, learning, and expertise development. This review has the potential to serve as a useful resource that will frame the current state of emerging evidence for health professions education researchers, while also identifying future directions for this exciting, novel approach.

## Materials and methods

We used a rigorous scoping review methodology that followed the recommendations provided by previous publications [7–9]. Scoping reviews, unlike systematic reviews, are not restricted to a specific set of questions and allow for a broader approach to examining the breadth, depth, and nature of research activity on an emerging research topic [8,10]. This type of review can help map a wide-ranging and heterogeneous literature base in order to summarize the current findings, identify the gaps, and inform future research endeavors [8].

Based on previous publications that provided guidance on scoping review methodology [7–9], our scoping review included five stages: (1) identifying the focused research question; (2) identifying the relevant studies; (3) selecting the studies to include; (4) charting the data; and (5) collating and reporting the results. Additionally, the Preferred Reporting Items for Systematic Reviews and Meta-Analyses (PRISMA) Extension for Scoping Reviews (PRISMA-ScR) guidelines [11] were used for reporting this study.

### Research question

We posed the following focused question to guide this scoping review: How has functional neuroimaging technology been used in health professions education to measure neurocognitive engagement, learning, and expertise development? Because a large literature base exists on diagnostic and clinical applications of functional neuroimaging, we intentionally kept the research question focused on learning and expertise development. However, this question was broadly inclusive of a wide spectrum of health professions learners from diverse clinical roles, disciplines, specialties, medical tasks, and settings. Our goal was not to summarize the rigor of existing research but to characterize the body of work on this emergent topic.

### Data sources and search strategy

One of the authors (J.N., a clinical informationist) developed the search strategy in collaboration with the rest of the team. The initial search was carried out on 4 April 2019, and then updated on 7 July 2020, in the following databases: Medline (PubMed), Embase (Embase.com), The Cochrane Library (Cochrane Database of Systematic Reviews), Cochrane Central Register of Controlled Trials (CENTRAL), Cochrane Methodology Register, ERIC (EbscoHost), and Web of Science (Science and Social Science Citation Index). When designing the search strategies for Medline (PubMed), the Cochrane Library, ERIC, and Embase, we identified controlled vocabulary terms for each concept and combined them with keyword synonyms. Web of Science was searched using keyword terms only.

Only articles in English were included owing to constraints related to resources for translating studies published in languages other than English (see Appendix 1 for exact search strategy). We also searched reference lists of review papers [2–6] and gray literature and identified six additional records from this process. We did not limit the results by publication date, as this is a rather new and emerging topic in health professions education.

### Screening and selecting the studies

Retrieved references were uploaded into the systematic review software Covidence (Veritas Health Innovation, Melbourne, Australia, available at www.covidence.org), which was used to remove duplicates and facilitate title, abstract, and full-text screening. Two of the authors (S.T. and D.S.) independently screened all of the retrieved titles and abstracts (a total of 1,524 records after duplicate removal) to determine their relevance for the research question and subsequent eligibility for full-text review. After screening the first 100 records, these authors met to discuss the discrepancies (only 11 at this point) and calibrate the screening process. The same two investigators independently completed the title and abstract screening with the remaining records. All of the disagreements (overall kappa 0.63) were resolved by consensus.

At the full-text screening stage, two authors (S.T. and D.S.) independently read all of the articles included for review and applied clear inclusion/exclusion criteria to determine eligibility. We included all English-language research and review articles that were published in peer-reviewed journals and considered the use of functional neuroimaging such as positron emission tomography (PET), electroencephalography (EEG), functional magnetic resonance imaging (fMRI), and functional near-infrared spectroscopy (fNIRS) in health professions education to measure neurocognitive engagement (e.g., attention, cognitive load), learning, and skill and expertise development. Studies were excluded based on the following criteria: focused on the clinical/diagnostic use of neuroimaging; involved patients in the use of neuroimaging; did not use neuroimaging for measuring some aspect of learning or skill development; did not involve medical trainees and/or health professionals. We also excluded editorials, commentaries, meeting abstracts, and articles published in a language other than English. The same two researchers reviewed any disagreements and reached a consensus on the final list of studies to be included in this review. As explained in the next stage, information from the included studies was recorded and summarized with a data charting template.

### Charting the data

The use of functional neuroimaging techniques to measure learning, neurocognitive engagement, or expertise development is an emerging research method in health professions education. The existing literature base is small and heterogeneous, making it challenging to draw specific comparisons between the different studies. Providing a brief summary for each of the records may not have much utility for informing future research either. Thus, we adopted a descriptive-analytical approach [8] by consistently applying a common analytical template to all included studies. This approach provided a useful framework for charting the pertinent information on research context and process.

We created a data entry template in Microsoft Excel 2016 (Redmond, Washington) to chart the key information from the studies included in this review. We sifted and sorted the pertinent information to help summarize and interpret the results, systematically charting the records in the process [8]. We then iteratively developed the data extraction sheet to capture relevant and consistent information from each study. Two of the authors (D.H. and J.M.) used this template independently to chart information from 10 randomly selected studies. During a meeting, the team went over these records and refined the template. Remaining articles were divided equally between the same two investigators to extract and chart data based on refined template criteria. To ensure completeness and accuracy, two additional investigators (S.T. and D.S.) divided all included studies equally and reviewed full-text articles against the charted records. The full team resolved any disagreements regarding the charted information by consensus. The extraction template included the following information

- Authors
- Year of publication
- Study location
- Study design
- Study aims
- Study population
- Tasks/intervention and duration
- Skill of interest (psychomotor, clinical reasoning, etc.)
- Neuroimaging modality
- Data acquisition, processing, and analysis
- Outcome measures
- Medical specialty
- Major findings

### Collating and summarizing the results

Charted data served as the basis for the numerical analysis of the range and nature of the included studies as well as for thematic outline of the findings. We organized the data to create a descriptive summary that encompassed the overall number of studies included; year of publication; country of origin; types of study design; medical specialty and/or study populations; skill(s) of interest; and types of neuroimaging modalities. To ensure appropriateness for interpretation, we also reorganized and further identified various themes that emerged from the extracted data to report common techniques used for neuroimaging data acquisition, processing, and analysis; neuroimaging modality use by year; skill of interest by use of neuroimaging modality; and skill of interest by medical specialty.

## Results

**Figure 1.**
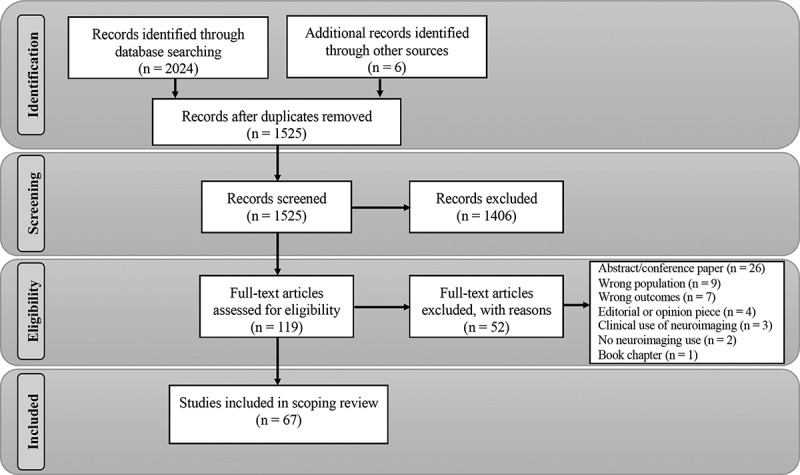
PRISMA flow diagram.

We retrieved 1,525 nonduplicate records for title and abstract screening; 119 met the eligibility criteria for full-text screening, and 67 met the final criteria as shown in Figure 1.

### Descriptive summary

The included articles (n = 67) were published between 2007 and 2020 [2–6,12–73]. Ninety percent of these publications (n = 60) were published in the last 10 years (2011–2020) [3–5,17–73], and 70% (n = 47), were published in the last 6 years (2015–2020) [3–5,30–73]. Although these studies were conducted in 16 different countries, the majority (68.7%, n = 46) were from three countries: USA (31.3%, n = 21), UK (22.4%, n = 15), and Canada (15%, n = 10). These publications came from 35 unique institutions (based on primary author affiliations). A total of 10 institutions had two or more articles, which represented 63% (n = 42) of all included articles.

Most of the articles were non-experimental (74.6%, n = 50) and were mainly exploratory in nature (feasibility, pilot, or proof-of-concept studies). The remaining articles were randomized controlled trials (11.9%, n = 8), review articles (7.5%, n = 5), randomized cross-over studies (3%, n = 2), and non-randomized controlled studies (3%, n = 2). Half of the non-experimental studies examined task and/or training effect in cohorts of novice learners (50%, n = 25/50), and a third used a between-subjects design to examine differences in the brain activation patterns either between novices under different conditions or between novices and experts (34%, n = 17/50). Some of the non-experimental studies were proof of concept (8%, n = 4/50), involving small numbers of participants, and others were cross-sectional (6%, n = 3/50). One study used a mixed-design approach by comparing different groups longitudinally (2%, n = 1/50).

As for the sample sizes of the included articles, several were feasibility studies and had five or fewer participants (n = 7), and the largest sample size was 62. Most of the included articles had 20 or fewer participants (54.8%, n = 34/62), whereas 21% (n = 13) had 21 to 30 and 24.2% (n = 15) had more than 31.

Most of the included studies were in surgery or surgical subspecialties (61.2%, n = 41). Other disciplines or learner groups included internal medicine (10.4%, n = 7), medical students (10.4%, n = 7), and gastroenterology (4.5%, n = 3). Four studies (6%) focused on various health sciences learners that did not fit into a single, specific learner group or specialty. There were also five single studies (each representing 1.5% of the total studies) in the following clinical specialties: anesthesiology, cardiology and pulmonology, pediatrics, psychiatry, and radiation oncology.

Most studies represented in this review investigated psychomotor skill development (57%, n = 38) and were conducted in the surgery specialties [13–15,17,20,21,23,28,30,33,34,36,38,39,43,48,49,51,55–58,61,63–67,70,71,74–76], but several (22%, n = 15) looked at neurocognitive correlates of clinical reasoning and diagnostic thinking [18,22,25,26,29,31,32,42,45–47,50,52,53,68]. A smaller number of studies (6%, n = 4) examined visuospatial expertise development [12,19,35,37]; declarative clinical knowledge [40,41,59,62] (6%, n = 4); and correlates of fatigue, well-being, and burnout [16,44,54,60] (6%, n = 4). Also, individual studies used neurocognitive imaging to examine cognitive and emotional control [27] (1.5%, n = 1), and crisis event management team skills [69] (1.5%, n = 1).

Included studies used three main neuroimaging modalities: fMRI (32.8%, n = 22), fNIRS (29.9%, n = 20), and EEG (26.8%, n = 18). The five review articles mentioned use of all these modalities. Summary characteristics for the included articles are shown in Table 1. Also, see Appendix 2 – Summary table listing the first author, publication year, country, primary author institution, study design, neuroimaging modality, specialty, and skill of interest for all included articles.

**Table 1.** Characteristics of articles included in the scoping review

Characteristic	N	Percent
**Year of publication**		
2007 to 2014	20	30
2015 to 2020	47	70
**Country of origin**		
Brazil	1	1.5
Canada	10	15.0
China	3	4.5
Denmark	1	1.5
Germany	1	1.5
Hong Kong	1	1.5
Ireland	2	3.0
Italy	2	3.0
Japan	4	6.0
Korea	1	1.5
Netherlands	2	3
Singapore	1	1.5
Spain	1	1.5
Turkey	1	1.5
UK	15	22.4
USA	21	31.3
**Study design**		
Non-experimental	50	74.6
Non-randomized controlled	2	3.0
Randomized crossover	2	3.0
Randomized controlled trial	8	11.9
Review	5	7.5
**Specialty/learner group**		
Anesthesiology	1	1.5
Cardiology & pulmonology	1	1.5
Gastroenterology	3	4.5
Internal medicine	7	10.4
Medical students	7	10.4
Health sciences learners	4	6.0
Pediatrics	1	1.5
Psychiatry	1	1.5
Radiation oncology	1	1.5
Surgery	41	61.2
**Skill of interest**		
Clinical reasoning	15	22.0
Cognitive/emotional control	1	1.5
Crisis event management	1	1.5
Clinical knowledge	4	6.0
Fatigue	2	3.0
Psychomotor skills	38	57.0
Well-being, stress, burnout	2	3.0
Visuospatial development	4	6.0
**Neuroimaging modality**		
EEG	18	26.8
fMRI	22	32.8
fNIRS/OT	20	29.9
Review – NA	5	7.5
Other*	2	3.0

EEG, electroencephalogram; fMRI, functional magnetic resonance imaging; fNIRS, functional near-infrared spectroscopy; NA, not applicable; OT, optical topography.

* Other included positron emission tomography (PET) [23] and a real time brain electric activity monitoring system developed by the researchers [30].

### Data acquisition, processing, and analysis

A detailed account of technical and methodological considerations for neuroimaging data acquisition and processing is beyond the scope of this review. Therefore, we briefly review some common steps involved in using this type of modality in health professions education research. Included research studies used various technology and software applications to acquire and process neuroimaging data based on the modality used (i.e., EEG, fMRI, fNIRS). The majority of the technologies used to acquire data were commercially available products, and few were developed at the researchers’ institution.

If researchers are going to use neuroimaging data as a continuous measure of task-related brain activation patterns, they must also ensure data quality and integrity when addressing the specific research question. For instance, some observed changes in brain activation patterns may be attributable to environmental factors or activities unrelated to the task in question. Thus, all of the studies used at least one software package along with filtering and classification techniques to remove noise and signal artifacts such as environmental or muscle and eye movement. The following are some of the software packages reported by the included research studies for neuroimaging data preprocessing and transformation: Analysis of Functional Neuroimages (AFNI) [77], fMRI Expert Analysis Tool (FEAT) [78,79], or FLIRT/MCFLIRT (Motion Correction FMRIB’s Linear Image Registration Tool) [80] for fMRI; HOMER2 [81], Functional optical signal analysis (fOSA) [82], or Imperial College Neuroimaging Analysis (ICNA) [83] or now known as Imperial College Near Infrared Spectroscopy Neuroimaging Analysis (ICNNA) [84] for fNIRS; and EEG lab [85] or Brain Vision Analyzer (Brain Products GmbH, Gilching, Germany) for EEG.

Once the results were filtered, processed, and transformed into a quantitative format, researchers used mainly general linear model approaches to examine the neuroimaging data. Correlation coefficients (Pearson or Spearman’s rank) and nonparametric tests were also used to analyze these data. A few studies (n = 4) specifically mentioned the use of machine learning algorithms [39,58,67,69].

### Neuroimaging modality use by year

**Figure 2.**
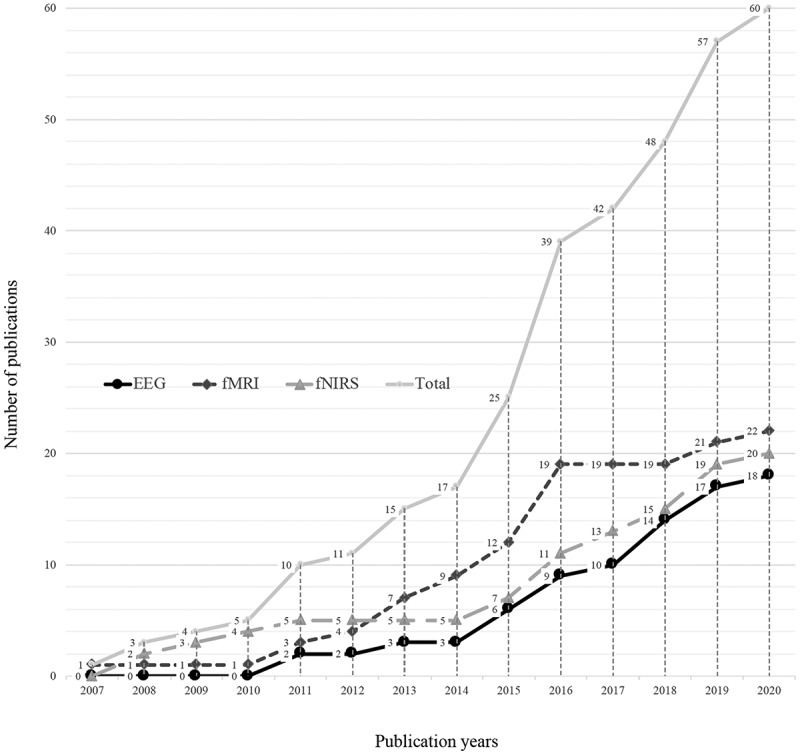
Cumulative number of studies that used each of the three main neuroimaging modalities by year. fMRI, functional magnetic resonance imaging; fNIRS, functional near-infrared spectroscopy; EEG, electroencephalography.

As described above, most of the research studies included in this review (97%, n = 60/62 [excluding review papers, n = 5]) used one of the three main neuroimaging modalities: fMRI, fNIRS, or EEG. Figure 2 shows the cumulative number of studies that used these three neuroimaging modalities across years. The number of published studies that used fMRI increased steadily between 2007 and 2016, but that increase has since slowed. In comparison, the number of fNIRS- and EEG-based studies has been stea7dily increasing since 2014.

### Skill of interest and modality

Most studies on psychomotor skill development used EEG (34%, n = 13/38) or fNIRS (32%, n = 12/38), and few used fMRI (16%, n = 6/38). In contrast, most of the studies that investigated clinical reasoning skills utilized fMRI technology (73.4%, n = 11/15). See Table 2 for the breakdown of studies in terms of skill of interest and neuroimaging modality.

**Table 2.** Skill of interest and modality

Skill	Modality					Total
	EEG	fMRI	fNIRS	Other*	Review	
Psychomotor skills	13 (34)	6 (16)	12 (32)	2 (5)	5 (13)	38
Clinical reasoning	2 (13.3)	11 (73.4)	2 (13.3)	–	–	15
Declarative clinical knowledge	2 (50)	2 (50)	–	–	–	4
Visuospatial skills	–	1 (25)	3 (75)	–	–	4
Fatigue	–	–	2 (100)	–	–	2
Well-being	1 (50)	1 (50)	–	–	–	2
Cognitive/emotional control	–	1 (100)	–	–	–	1
Crisis event management	–	–	1 (100)	–	–	1

All data are shown as n (%).

Abbreviations: EEG, electroencephalogram; fMRI, functional magnetic resonance imaging; fNIRS, functional near-infrared spectroscopy.

* Other included positron emission tomography (PET) [23] and a real time brain electric activity monitoring system developed by the researchers [30].

### Skill of interest by specialty

Studies that examined psychomotor skill development and visuospatial skills were conducted predominantly in surgery or surgical subspecialties (95%, n = 36/38; 75%, n = 3/4, respectively), whereas those focused on clinical reasoning were conducted mainly in internal medicine (47%, n = 7/15). Studies that examined declarative clinical knowledge utilized medical and health sciences students (100%, n = 4).

## Discussion

The purpose of this scoping review was to assess the existing literature related to functional neuroimaging of neurocognitive engagement in health professions education. Since the focus in this review was on the use of neuroimaging to study learning, we did not include interventional applications of neuroimaging such as neurofeedback. We also did not include studies that used EEG primarily as a measure of fatigue or sleep deprivation. However, if a study question focused on the effect of EEG measured sleep deprivation on learning, we did include the study. This review is the first to map the relevant literature on this topic. Synthesizing the research findings and indicating the untapped potentials of neuroimaging use could raise awareness, inform future efforts in this line of inquiry, and stimulate more interest in conducting such research in health professions education.

Our findings show increasing reports of studies, especially in the last 5 years, and likely enhanced momentum, as medical educators recognize the opportunities to provide objective evidence. The rapid development of user-friendly technologies, such as wireless EEG and fNIRS headsets, and increased access to fMRI scanners also likely contributes to the increased pace. Recognizing the accessibility of these new technologies and their applications for learning may pave the way for future studies.

Our results show patterns of use that are related to either date of study or study question. The earlier studies, 2007–2014, primarily utilized fMRI, and the later studies, 2015–2020, show increasing use of EEG and fNIRS. We believe that this progression is due to the increased availability of easier-to-use wireless headsets and software for data acquisition and analysis. These changes also coincided with the rapid expansion of large-database use in medical science. Each of the imaging modalities creates massive amounts of data. Therefore, current and future considerations for regular use of neuroimaging in assessment of learning must include plans for data storage, data management, and accuracy of software algorithms.

Notably, research questions can lend themselves to one imaging modality or another. Both fMRI and fNIRS are brain mapping techniques that rely on hemodynamic response for capturing neurocognitive processes. fMRI provides high spatial resolution for exploring activation patterns of specific brain regions associated with various cognitive tasks. When the study goal is to look for temporal effects, fNIRS is better than fMRI. However, both have relatively low temporal resolution compared to EEG, which offers temporal resolution on the order of milliseconds for examining task-related brain activation patterns. See Table 3 for more comparisons between neuroimaging modalities. Some groups have explored options of combining measurement modalities to optimize temporal and spatial resolution however logistical impediments remain an important barrier.

**Table 3.** Characteristics of neuroimaging modalities

Modality	How it works	Best applications	Advantages [86]	Disadvantages [86]
fMRI	Captures hemodynamic response/regional activated neuroanatomy	Clinical reasoning Declarative knowledge	High spatial resolution (3–4 mm) Can assess deep brain regions	Cost Not usable in clinical environment possible false positive rate longer temporal resolution 3–6 sec)
fNIRS	Captures hemodynamic response through optical intensity/regional brain blood flow	Psychomotor skills	Intermediate temporal resolution (3–6 sec) portable	Limited to superficial brain regions Lower spatial resolution (10–20 mm)
EEG	Electrical activity of the brain showing wavelength and areas of activation Event-related potentials	Psychomotor skills	High temporal resolution (1–100 ms) Portable Lowest cost	Lower spatial resolution (10–20 mm)

Abbreviations: EEG, electroencephalogram; fMRI, functional magnetic resonance imaging; fNIRS, functional near-infrared spectroscopy.

Our results show that the literature base on this topic is still emerging and that the majority of the studies are exploratory (feasibility, pilot, or proof-of-concept). The heterogeneity of the target learner populations, research questions, study designs, targeted skills, interventions, outcome measures, and neuroimaging modalities used makes it impractical to systematically compare outcomes across studies. However, our review of the main results identified a few common themes within the included studies. The researchers either stated explicitly or indicated implicitly (by the study design) that they adopted information processing, neural efficiency, and/or dual processing theory to inform their research.

These frameworks within the context of these studies help explain differences in the brain activation patterns between novices and experts. Evidence from cognitive neuroscience has shown that the frontal cortex is a major hub for storage and executive processes [87]. Individuals are thought to better regulate their working memory resources and exhibit significantly less brain activation in the prefrontal cortex (PFC) while performing tasks within their area of expertise [88]. Similarly, dual processing theory suggests that with experience and proficiency, slow, analytical decision-making evolves into less-effortful, intuitive judgements [89].

Overall, included research studies that used functional neuroimaging technology during technical skill performance indicated that experts and novices show distinct brain activation patterns. Experts show significantly lower activation, especially in the PFC, than do novices. One study with a longitudinal component found that after repeated practice, novices still showed persistent engagement in the PFC even after they demonstrated expert level performance based on observational measures [51]. This finding may in fact have some implications for clinical educators and hint that demonstrated competency in a controlled educational environment may not necessarily translate into complex clinical settings.

Complex procedural skills that require coordination of multiple sensorimotor functions or pathways may not be detected clearly enough in the brain activation patterns to distinguish skill levels. For instance, one included study hypothesized that functional connectivity data within sensorimotor and associative networks may be a better measure to appreciate differences in surgeon skill levels [39]. This group applied machine learning algorithms to functional brain connectivity data and was able to classify surgeon skill levels with good accuracy. Their findings are still in line with other included studies that reported increased PFC activation in the novices but also adds that novices showed stronger interaction between prefrontal and motor-related regions than did experts.

Although high levels of brain activation in the PFC could hinder performance, low levels of activation might indicate lack of attention and concentration. For example, one group examined the effect of temporal pressure on laparoscopic suturing performance and on the neurocognitive correlates of task load (Modi et al., 2018). Performance scores decreased for all trainees under time-pressure conditions when compared to self-pacing. All trainees reported greater task load under time pressure and showed decreased PFC activity, especially junior trainees. In a subsequent study, this group used fNIRS to examine brain activation differences between trainees who maintained a stable performance under time pressure and those who did not [65]. Resilient trainees showed greater bilateral ventral PFC activation, suggesting better attentional control and vigilance. Interestingly, researchers found that stress sensitivity and performance degradation were unrelated to experience level and were not captured by the heart-rate variability or self-report work-load measures (Surgical-TLX). The current literature base does not provide clear markers for the optimal PFC engagement levels, which may be dependent on the context, task, and individual.

Similarly, studies examining clinical reasoning or decision-making indicated that experts and novices show different brain activation patterns, especially, in the PFC. Additionally, the nature of the task seems to modulate the brain activation patterns, as several studies showed that problem-solving tasks or difficult cases induced significantly higher brain activation than did simple recall or routine clinical cases [25,42,46,52,68]. Several of these clinical decision-making studies specifically examined whether dual processing theory could be verified by the use of functional brain imaging [46,47,68]. A few studies also looked at the changes in brain activation patterns as a function of learning or based on high or low performance [18,22,27,40,45]. For example, one group showed that incorrect answers induced higher PFC activation than did correct answers [22].

This review also indicates a few areas ripe for further research efforts. One such area is the establishment of a viable study design and research methods for operational settings. Though researchers attempted to approximate real-life tasks, studies embedded in operational settings with realistic clinical tasks are scarce. For environmental validity, future research endeavors may use real-world settings, perhaps by integrating functional neuroimaging within simulated clinical environments. For example, one study included in this review explored the use of functional neuroimaging to examine team functioning [69] during high-fidelity simulation-based training for crisis event management in the operating rooms. This group showed that it was feasible to use wireless functional neuroimaging technology to measure workload and team engagement in simulated clinical settings. Their results suggested that deoxyhemoglobin in the PFC was a good indicator of workload for individuals but that oxyhemoglobin neural synchrony was more sensitive to scenario difficulty levels. The teams that participated in that study showed greater neural synchrony during teamwork. These findings are encouraging; however, team dynamics in fluid clinical settings are quite complex, and more work is needed to continue building evidence for reliability and validity for capturing neurocognitive correlates of team experience.

Our review did not identify a strong literature base within the health professions education for real-time monitoring of tasks that require vigilance and sustained attention. One group examined the effect of fatigue on clinical reasoning skills across two separate pilot studies as internal medicine physicians answered and reflected on multiple-choice questions from the US medical licensing and/or maintenance of certification exams [29,32]. The results were consistent with those of other studies outside medical education, which have shown that fatigue and sleep quantity are associated with significant changes in brain activation patterns, especially in the medial and/or lateral PFC and other working memory-related areas. Although the exact nature of this relationship was not clear, these preliminary findings emphasize the importance of examining how fatigue and sleep deprivation might regulate neurocognitive engagement. The interplay among various factors such as expertise level, task, cognitive load, attention, and medical errors presents critical research opportunities for functional neuroimaging studies. Numerous studies in other fields could offer guidance for such efforts in health professions education. This type of research can have significant implications for real-time monitoring, and potentially intervention, during critical clinical tasks.

Several studies have used learning curves to visualize learning/competency development in various clinical skills as a function of effort and accuracy/achievement plotted across multiple data points [90]. However, studies that use functional neuroimaging to track and verify the expertise development process are lacking. Future longitudinal studies that examine changes in the brain activation patterns of learners could be invaluable to understanding the learning process at a more individual level and for potentially devising ways to individualize and optimize health professions training. These research opportunities are vast, and the impact of this approach to health professions education is important, as it may provide novel and complementary measures of learning and perhaps additional insights into competency development from a neurocognitive perspective.

Another question ripe for study is whether gender differences in brain activation patterns appear in studies of this type. Two articles [24,71] specifically mentioned comparing brain activation patterns between male and female participants. Both studies examined laparoscopic skill development. Bocci et al. (2013) used EEG to measure the differences in hemispheric connectivity in surgeons using either laparoscopic or robotic surgery for the same motor task. This study did not find any significant differences between males and females performing the same task in neither hemispheres [24]. The second study [71], a randomized controlled trial, used fNIRS to assess the effects of standardized laparoscopic workshops on medical students’ prefrontal cortex activation (PFCA) patterns. Overall, trained individuals showed left PFCA attenuation for precision cutting task. Subgroup analyses indicated that trained female students had significantly greater PFCA attenuation compared to untrained female students for peg transfer and precision cutting tasks whereas no difference was noted between trained and untrained males. The researchers concluded that female students were better able to focus and thus benefitted from focused laparoscopic training. However, these differences in PFCA were not reflected in objective performance scores.

We currently do not have efficient methods for gaining a full understanding of the neurocognitive engagement of learners. Educational efficacy could be markedly augmented if one were able to recognize when cognitive load increases enough to impair attention, when situational awareness is impaired, or when fatigue reduces attention. Understanding such neurocognitive engagement of the learner in the classroom and particularly at the bedside may facilitate learning and possibly enhance patient safety. In an age when learner-centered approaches, much like patient-centered medicine, have recognized value, we must develop efficient and comprehensive measures of learning success across various classroom and clinical educational settings. Recognition of impaired learning may help programs make adjustments to instructional design, learning environment, pace, or repetition.

Finally, we were not able to find strong presence of power and sample size considerations in the included studies. As reported earlier, most of the studies had considerably small sample sizes. The fact that neuroimaging provides rich data may allow for the detection of meaningful differences in the brain activation patterns. However, as this research matures, it will be important to provide robust methodology for sample size considerations with respect to the research question. This field of study will be optimally developed with more longitudinal, comprehensive and hypothesis-driven investigations. We need to learn more about brain dynamics in both learners and experts and how neuroimaging modalities can capture those differences. Additionally, technological development of sophisticated and affordable wearable devices should improve the possibilities of using neuroimaging in live, patient care environments.

Limitations to reviews of this nature include the possibility of bias in selection of the published articles. We limited our selection committee to two investigators after establishing our research question and search methods and conducted a calibration exercise at the beginning of screening. Each investigator screened the candidate studies for inclusion, and discrepancies were discussed and resolved. PRISMA-ScR guidelines were followed in an attempt to generate a transparent report of the available literature.

## Conclusions

This scoping review provides an overview of the available literature on the topic of neuroimaging use for health professions educational research. Neuroimaging has the potential to help validate/challenge established theoretical assumptions, lay foundations for new theory development, and provide insight into more efficient and efficacious training methods in health professions education. The use of these emerging technologies and databases appears ripe for the development of precision education and for understanding the learning curve of each individual learner in specific skills or cognitive engagement. Opportunities may also exist for establishing objective measures of competence.

## AppendicesAppendix 1- Search Strategy conducted on 4/4/2019 and updated on 7/7/2020

**Table:**

**PubMed**	
#1	(‘Neuroimaging’[Mesh] OR ‘Electroencephalography’[Mesh] OR FMRI[tiab] OR EEG[tiab] OR ERP[tiab] OR fnirs[tiab] OR neuroimag*[tiab] OR ‘brain imaging’[tiab] OR ‘functional mri’[tiab] OR ‘functional magnetic resonance spectroscopy’[tiab] OR electroencephalography[tiab] OR ‘event related potentials’[tiab] OR ‘functional neuroimaging’[tiab] OR ‘Functional near infrared spectroscopy’[tiab])
_#2_	(‘Clinical Competence’[Mesh] OR ‘Psychomotor Performance’[Mesh] OR ‘Burnout, Psychological’[Mesh] OR ‘Burnout, Professional’[Mesh] OR ‘Sleepiness’[Mesh] OR ‘cognitive load’[tiab] OR clinical skill*[tiab] OR ‘clinical expertise’[tiab] OR ‘skills learning’[tiab] OR ‘skill acquisition’[tiab] OR surgical skill*[tiab] OR ‘clinical reasoning’[tiab] OR psychomotor skill*[tiab] OR procedural skill*[tiab] OR technical skill*[tiab] OR ‘diagnostic thinking’[tiab] OR ‘diagnostic reasoning’[tiab] OR ‘visual expertise’[tiab] OR burnout[tiab] OR sleepiness[tiab] OR fatigue[tiab] OR performance[tiab] OR competenc*[tiab] OR ‘mental workload’[tiab] OR memory[tiab] OR ‘problem solving’[tiab] OR medical error*[tiab] OR ‘patient safety’[tiab])
#3	(‘Surgeons’[MeSH] OR ‘Physicians’[MeSH] OR ‘Students, Medical’[Mesh] OR ‘Internship and Residency’[Mesh] OR ‘Clinical Clerkship’[Mesh] OR ‘premedical’[tiab] OR ‘pre-medical’[tiab] OR ‘clinical clerkship’[tiab] OR ‘resident’[tiab] OR ‘residents’[tiab] OR ‘residency’[tiab] OR PGY*[tiab] OR ‘post graduate year’[tiab] OR ‘postgraduate year’[tiab] OR ‘trainee’[tiab] OR ‘intern’[tiab] OR ‘interns’[tiab] OR ‘internship’[tiab] OR ‘house officer’[tiab] OR ‘house officers’[tiab] OR ‘houseofficer’[tiab] OR ‘house staff’[tiab] OR ‘housestaff’[tiab] OR ‘fellowship’[tiab] OR ‘fellow’[tiab] OR ‘fellows’[tiab] OR ‘undergraduate medical’[tiab] OR ‘medical student’[tiab] OR ‘medical students’[tiab] OR ‘Schools, Medical’[Mesh] OR medical school*[tiab] OR health science*[tiab] OR clinician*[tiab] OR surgeon*[tiab] OR physician[tiab] OR physicians[tiab] OR ‘health profession’[tiab] OR ‘health professionals’[tiab] OR ‘health professions’[tiab] OR doctor[tiab] OR doctors[tiab])
#4	(‘Education, Medical’[Mesh] OR ‘Education’[Mesh] OR educate[tiab] or education[tiab] OR educated[tiab] OR educational[tiab] OR curricula*[tiab] OR curriculum[tiab] OR train*[tiab] OR teach*[tiab] OR instruct*[tiab] OR mentor*[tiab] OR program*[tiab] OR pedagog*[tiab] OR workshop*[tiab] OR initiative*[tiab] OR seminar*[tiab] OR electives[tiab] OR ‘CME’[tiab] OR ‘faculty development’[tiab])
#5	#1 AND #2 AND #3 AND #4
**Cochrane**	
#1	(‘Neuroimaging’[Mesh] OR ‘Electroencephalography’[Mesh] OR FMRI OR EEG OR ERP OR fnirs OR neuroimag* OR ‘brain imaging’ OR ‘functional mri’ OR ‘functional magnetic resonance spectroscopy’ OR electroencephalography OR ‘event related potentials’ OR ‘functional neuroimaging’ OR ‘Functional near infrared spectroscopy’)
#2	(‘Clinical Competence’[Mesh] OR ‘Psychomotor Performance’[Mesh] OR ‘Burnout, Psychological’[Mesh] OR ‘Burnout, Professional’[Mesh] OR ‘Sleepiness’[Mesh] OR ‘cognitive load’ OR clinical skill* OR ‘clinical expertise’ OR ‘skills learning’ OR ‘skill acquisition’ OR surgical skill* OR ‘clinical reasoning’ OR psychomotor skill* OR procedural skill* OR technical skill* OR ‘diagnostic thinking’ OR ‘diagnostic reasoning’ OR ‘visual expertise’ OR burnout OR sleepiness OR fatigue OR performance OR competenc* OR ‘mental workload’ OR memory OR ‘problem solving’ OR medical error* OR ‘patient safety’)
#3	(‘Surgeons’[MeSH] OR ‘Physicians’[MeSH] OR ‘Students, Medical’[Mesh] OR ‘Internship and Residency’[Mesh] OR ‘Clinical Clerkship’[Mesh] OR ‘premedical’ OR ‘pre-medical’ OR ‘clinical clerkship’ OR ‘resident’ OR ‘residents’ OR ‘residency’ OR PGY* OR ‘post graduate year’ OR ‘postgraduate year’ OR ‘trainee’ OR ‘intern’ OR ‘interns’ OR ‘internship’ OR ‘house officer’ OR ‘house officers’ OR ‘houseofficer’ OR ‘house staff’ OR ‘housestaff’ OR ‘fellowship’ OR ‘fellow’ OR ‘fellows’ OR ‘undergraduate medical’ OR ‘medical student’ OR ‘medical students’ OR ‘Schools, Medical’[Mesh] OR medical school* OR health science* OR clinician* OR surgeon* OR physician OR physicians OR ‘health profession’ OR ‘health professionals’ OR ‘health professions’ OR doctor OR doctors)
#4	(‘Education, Medical’[Mesh] OR ‘Education’[Mesh] OR educate or education OR educated OR educational OR curricula* OR curriculum OR train* OR teach* OR instruct* OR mentor* OR program* OR pedagog* OR workshop* OR initiative* OR seminar* OR electives OR ‘CME’ OR ‘faculty development’)
#5	#1 AND #2 AND #3 AND #4
**Web of Science**	
#1	TS = (FMRI OR EEG OR ERP OR fnirs OR neuroimag* OR ‘brain imaging’ OR ‘functional mri’ OR ‘functional magnetic resonance spectroscopy’ OR electroencephalography OR ‘event related potentials’ OR ‘functional neuroimaging’ OR ‘Functional near infrared spectroscopy’)
#2	TS = (‘cognitive load’ OR clinical skill* OR ‘clinical expertise’ OR ‘skills learning’ OR ‘skill acquisition’ OR surgical skill* OR ‘clinical reasoning’ OR psychomotor skill* OR procedural skill* OR technical skill* OR ‘diagnostic thinking’ OR ‘diagnostic reasoning’ OR ‘visual expertise’ OR burnout OR sleepiness OR fatigue OR performance OR competenc* OR ‘mental workload’ OR memory OR ‘problem solving’ OR medical error* OR ‘patient safety’)
#3	TS = (‘premedical’ OR ‘pre-medical’ OR ‘clinical clerkship’ OR ‘resident’ OR ‘residents’ OR ‘residency’ OR PGY* OR ‘post graduate year’ OR ‘postgraduate year’ OR ‘trainee’ OR ‘intern’ OR ‘interns’ OR ‘internship’ OR ‘house officer’ OR ‘house officers’ OR ‘houseofficer’ OR ‘house staff’ OR ‘housestaff’ OR ‘fellowship’ OR ‘fellow’ OR ‘fellows’ OR ‘undergraduate medical’ OR ‘medical student’ OR ‘medical students’ OR ‘medical school*’ OR ‘health science*’ OR clinician* OR surgeon* OR physician OR physicians OR ‘health profession’ OR ‘health professionals’ OR ‘health professions’ OR doctor OR doctors)
#4	TS = (educate OR education OR educated OR educational OR curricula* OR curriculum OR train* OR teach* OR instruct* OR mentor* OR program* OR pedagog* OR workshop* OR initiative* OR seminar* OR electives OR CME OR ‘faculty development’)
#5	#1 AND #2 AND #3 AND #4
**Embase**	
#1	‘neuroimaging’/exp OR ‘electroencephalography’/exp OR (FMRI OR EEG OR ERP OR fnirs OR neuroimag* OR ‘brain imaging’ OR ‘functional mri’ OR ‘functional magnetic resonance spectroscopy’ OR electroencephalography OR ‘event related potentials’ OR ‘functional neuroimaging’ OR ‘Functional near infrared spectroscopy’):ti,ab
#2	‘clinical competence’/exp OR ‘psychomotor performance’/exp OR ‘burnout’/exp OR (‘cognitive load’ OR ‘clinical skill*’ OR ‘clinical expertise’ OR ‘skills learning’ OR ‘skill acquisition’ OR ‘surgical skill*’ OR ‘clinical reasoning’ OR ‘psychomotor skill*’ OR ‘procedural skill*’ OR ‘technical skill*’ OR ‘diagnostic thinking’ OR ‘diagnostic reasoning’ OR ‘visual expertise’ OR burnout OR sleepiness OR fatigue OR performance OR competenc* OR ‘mental workload’ OR memory OR ‘problem solving’ OR ‘medical error*’ OR ‘patient safety’):ti,ab
#3	‘medical education’/exp OR ‘surgeon’/exp OR ‘physician’/exp OR ‘health student’/exp OR ‘resident’/exp OR (premedical OR pre-medical OR ‘clinical clerkship’ OR resident OR residents OR residency OR PGY* OR ‘post graduate year’ OR ‘postgraduate year’ OR trainee OR intern OR interns OR internship OR ‘house officer’ OR ‘house officers’ OR ‘houseofficer’ OR ‘house staff’ OR housestaff OR fellowship OR fellow OR fellows OR ‘undergraduate medical’ OR ‘medical student’ OR ‘medical students’ OR ‘medical school*’ OR ‘health science*’ OR clinician* OR surgeon* OR physician OR physicians OR ‘health profession’ OR ‘health professionals’ OR ‘health professions’ OR doctor OR doctors):ti,ab
#4	‘medical education’/exp OR ‘education’/exp OR (educate OR education OR educated OR educational OR curricula* OR curriculum OR train* OR teach* OR instruct* OR mentor* OR program* OR pedagog* OR workshop* OR initiative* OR seminar* OR electives OR CME OR ‘faculty development’):ti,ab
#5	#1 AND #2 AND #3 AND #4
**ERIC**	
#1	(FMRI OR EEG OR ERP OR fnirs OR neuroimag* OR ‘brain imaging’ OR ‘functional mri’ OR ‘functional magnetic resonance spectroscopy’ OR electroencephalography OR ‘event related potentials’ OR ‘functional neuroimaging’ OR ‘Functional near infrared spectroscopy’)
#2	(DE ‘Medical Evaluation’) OR (DE ‘Psychomotor Skills’) OR (DE ‘Burnout’) OR ‘cognitive load’ OR ‘clinical skill*’ OR ‘clinical expertise’ OR ‘skills learning’ OR ‘skill acquisition’ OR ‘surgical skill*’ OR ‘clinical reasoning’ OR ‘psychomotor skill*’ OR ‘procedural skill*’ OR ‘technical skill*’ OR ‘diagnostic thinking’ OR ‘diagnostic reasoning’ OR ‘visual expertise’ OR burnout OR sleepiness OR fatigue OR performance OR competenc* OR ‘mental workload’ OR memory OR ‘problem solving’ OR ‘medical error*’ OR ‘patient safety’)
#3	(DE ‘Medical Education’) OR (DE ‘Medical Schools’) OR (DE ‘Physicians’) OR (DE ‘Medical Students’) OR ‘premedical’ OR ‘pre-medical’ OR ‘clinical clerkship’ OR resident OR residents OR residency OR PGY* OR ‘post graduate year’ OR ‘postgraduate year’ OR trainee OR intern OR interns OR internship OR ‘house officer’ OR ‘house officers’ OR houseofficer OR ‘house staff’ OR housestaff OR fellowship OR fellow OR fellows OR ‘undergraduate medical’ OR ‘medical student’ OR ‘medical students’ OR ‘medical school*’ OR ‘health science*’ OR clinician* OR surgeon* OR physician OR physicians OR ‘health profession’ OR ‘health professionals’ OR ‘health professions’ OR doctor OR doctors)
#4	(DE ‘Medical Education’) OR (DE ‘Education’) OR educate or education OR educated OR educational OR curricula* OR curriculum OR train* OR teach* OR instruct* OR mentor* OR program* OR pedagog* OR workshop* OR initiative* OR seminar* OR electives OR ‘CME’ OR ‘faculty development’)
#5	#1 AND #2 AND #3 AND #4

## Appendix 2: Summary table for included studies in alphabetical order by first author

**Table:**

Authors	Year	**Country**	Primary Author Institution	Design	Neuroimaging Modality	Specialty	Skill
Aggarwal et al.	2008	**UK**	Department of Biosurgery & Surgical Technology, Imperial College London	Review	N/A	surgery	REVIEW psychomotor skill
Akimoto et al.	2015	**Japan**	Center for Advanced Medical Initiatives, Kyushu University	Non-experimental	Other *	surgery	psychomotor skill
Aksoy et al.	2019	**Turkey**	Department of Biomedical Device Technology, Acıbadem Mehmet Ali Aydınlar University	Non-experimental	fNIRS	healthcare professionals	psychomotor skill
Anderson et al.	2019	**Canada**	Department of Community Health Sciences, Cumming School of Medicine, University of Calgary	RCT	EEG	health sciences students	declarative clinical knowledge
Andreu-Perez et al.	2016	**UK**	The Hamlyn Centre, Imperial College London	Non-experimental	fNIRS/OT	surgery	psychomotor skill
Bahrami et al.	2014	**Canada**	Institute of Biomaterials and Biomedical Engineering, University of Toronto	Non-experimental	fMRI	surgery	psychomotor skill
Bahrami et al.	2011	**Canada**	Institute of Biomaterials and Biomedical Engineering, University of Toronto, and Keenan Research Centre of the Li Ka Shing Knowledge Institute at St. Michael’s Hospital, Toronto	Non-experimental	fMRI	surgery	psychomotor skill
Bernier et al.	2016	**Canada**	Sherbrooke University Hospital Center (CHUS)	Non-experimental	fMRI	medical students	declarative clinical knowledge
Bhatt et al.	2016	**Ireland**	Department of Surgery, University of Dublin, Trinity College, at the Adelaide and Meath Hospital	Review	N/A	surgery	REVIEW effects of aging on surgeons
Bocci et al.	2013	**Italy**	Department of Neuroscience, Unit of Neurology, Pisa University Medical School	Non-experimental	EEG	surgery	psychomotor skill
Brod et al.	2016	**Germany**	The Center for Lifespan Psychology, Max Planck Institute for Human Development	Non-experimental	fMRI	medical students	declarative clinical knowledge
Chang et al.	2016	**South Korea**	Department of Medical Humanities, Korea University College of Medicine	Non-experimental	fMRI	medical students	clinical reasoning
Ciechanski et al.	2019	**Canada**	Faculty of Medicine and Dentistry, University of Alberta	RCT	EEG	surgery	psychomotor skill
Crewther et al.	2016	**UK**	The Hamlyn Centre for Robotic Surgery, Imperial College London	Non-experimental	fNIRS	surgery	psychomotor skill
De Andrade et al.	2016	**Brazil**	Faculty of Medicine, Department of Paediatric, University of Sao Paulo	Non-experimental	fMRI	pediatrics	well-being
Downar et al.	2011	**USA**	Human Neuroimaging Laboratory, Virginia Tech Carilion Research Institute and Department of Physics, Virginia Tech	Non-experimental	fMRI	non-surgical physicians	clinical reasoning
Durning et al.	2013	**USA**	Department of Medicine, Uniformed Services University of the Health Sciences	Non-experimental	fMRI	internal medicine	clinical reasoning
Durning et al.	2014	**USA**	Department of Medicine, Uniformed Services University of the Health Sciences	Non-experimental	fMRI	internal medicine	clinical reasoning
Durning et al.	2015	**USA**	Department of Medicine, Uniformed Services University of the Health Sciences	Non-experimental	fMRI	internal medicine	clinical reasoning
Durning et al.	2016	**USA**	Department of Medicine, Uniformed Services University of the Health Sciences	Non-experimental	fMRI	internal medicine	clinical reasoning
Durning et al.	2013	**USA**	Department of Medicine, Uniformed Services University of the Health Sciences	Non-experimental	fMRI	internal medicine	clinical reasoning
Durning et al.	2012	**USA**	Department of Medicine, Uniformed Services University of the Health Sciences	Non-experimental	fMRI	internal medicine	clinical reasoning
Durning et al.	2015	**USA**	Department of Medicine, Uniformed Services University of the Health Sciences	Non-experimental	fMRI	internal medicine	clinical reasoning
Duty et al.	2012	**USA**	Institute for Urology and Center of Neurosciences, Feinstein Institute for Medical Research North Shore–Long Island Jewish Health System	Non-experimental	PET	surgery	psychomotor skill
Garbens et al.	2019	**Canada**	Department of Surgery, St. Michael’s Hospital, University of Toronto	Non-experimental	fMRI	surgery	psychomotor skill
Guru et al.	2015	**USA**	Department of Urology, Roswell Park Cancer Institute, Brain Computer Interface Laboratory, Department of Mechanical & Aerospace Engineering, University at Buffalo	Non-experimental	EEG	surgery	psychomotor skill
Guru et al.	2015	**USA**	Department of Urology, Applied Technology Laboratory for Advanced Surgery (ATLAS) Program at Roswell Park Cancer Institute	Non-experimental	EEG	surgery	psychomotor skill
Hruska et al.	2015	**Canada**	Department of Community Health Sciences, Cumming School of Medicine, University of Calgary	Non-experimental	fMRI	gastroenterology	diagnostic thinking
Hruska et al.	2015	**Canada**	Department of Community Health Sciences, Cumming School of Medicine, University of Calgary	Non-experimental	fMRI	gastroenterology	diagnostic thinking
Hussein et al.	2016	**USA**	Applied Technology Laboratory for Advanced Surgery (ATLAS) Program, Roswell Park Cancer Institute, Buffalo	Non-experimental	EEG	surgery	psychomotor skill
James et al.	2011	**UK**	Department of Surgery and Cancer & Hamlyn Centre for Robotic Surgery, Imperial College	Non-experimental	fNIRS	gastroenterology	visuospatial skill
Kahol et al.	2011	**USA**	Human Machine Symbiosis Laboratory, School of Biological and Health Systems Engineering, Arizona State University	Non-experimental	EEG	surgery	psychomotor skill
Karabanov et al.	2019	**Denmark**	Danish Research Centre for Magnetic Resonance, Centre for Functional and Diagnostic Imaging and Research, Copenhagen University Hospital	RCT	fMRI	surgery	psychomotor skill
Khoe et al.	2020	**Singapore**	Yong Loo Lin School of Medicine, National University of Singapore	RCT	fNIRS	surgery	psychomotor skill
Leff et al.	2008	**UK**	Royal Wolfson Image Computing Laboratory and Department of Biosurgery and Surgical Technology, Imperial College London	Non-experimental	fNIRS	surgery	psychomotor skill
Leff et al.	2015	**UK**	Hamlyn Centre for Robotic Surgery, Imperial College London	Randomized crossover	fNIRS/OT	surgery	visuospatial skill
Leff et al.	2008	**UK**	Royal Wolfson Image Computing Laboratory and Department of Biosurgery and Surgical Technology, Imperial College London	Review	N/A	surgery	REVIEW psychomotor skill
Leff et al.	2008	**UK**	Royal Wolfson Image Computing Laboratory and Department of Biosurgery and Surgical Technology, Imperial College London	Non-experimental	fNIRS	surgery	psychomotor skill
Leff et al.	2010	**UK**	Royal Wolfson Image Computing Laboratory and Department of Biosurgery and Surgical Technology, Imperial College London	Non-experimental	fNIRS	surgery	fatigue
Leff et al.	2017	**UK**	Hamlyn Centre for Robotic Surgery, Department of Biosurgery and Surgical Technology, Imperial College London	Non-experimental	fNIRS/OT	surgery	clinical reasoning
Li et al.	2020	**China**	Spinal Division of Orthopedic and Traumatology Center, Affiliated Hospital of Guangdong Medical University	Non-experimental	EEG	surgery	psychomotor skill
Maddox et al.	2015	**USA**	Department of Urology, Tulane University School of Medicine	Non-experimental	EEG	surgery	psychomotor skill
Mazur et al.	2017	**USA**	Department of Radiation Oncology, University of North Carolina	Non-experimental	EEG	radiation oncology	clinical reasoning
Mazur et al.	2016	**USA**	Department of Radiation Oncology, University of North Carolina	Non-experimental	EEG	medical trainees	psychomotor skill
Miura et al.	2015	**Japan**	Department of Modern Mechanical Engineering, Waseda University	Non-experimental	fNIRS	surgery	visuospatial skill
Modi et al.	2019	**UK**	Department of Surgery and Cancer & Hamlyn Centre for Robotic Surgery, Imperial College London	Non-experimental	fNIRS	surgery	psychomotor skill
Modi et al.	2018	**UK**	Department of Surgery and Cancer & Hamlyn Centre for Robotic Surgery, Imperial College London	Non-experimental	fNIRS	surgery	psychomotor skill
Modi et al.	2017	**UK**	Department of Surgery and Cancer & Hamlyn Centre for Robotic Surgery, Imperial College London	Review	N/A	surgery	REVIEW psychomotor skill
Modi et al.	2017	**UK**	Department of Surgery and Cancer & Hamlyn Centre for Robotic Surgery, Imperial College London	Review	N/A	surgery	REVIEW psychomotor skill
Morales et al.	2019	**Spain**	Mind, Brain, and Behavior Research Center, University of Granada	Non-experimental	EEG	surgery	psychomotor skill
Morris et al.	2015	**Ireland**	Education Division and Department of Surgery, School of Medicine, Trinity College	Non-experimental	fMRI	surgery	psychomotor skill
Ndaro et al.	2018	**China**	Department of Biomedical Engineering, School of Medical Instrument and Food Engineering, University of Shanghai for Science and Technology	Non-experimental	EEG	surgery	psychomotor skill
Nemani et al.	2018	**USA**	Rensselaer Polytechnic Institute	RCT	fNIRS	surgery	psychomotor skill
Nishida et al.	2017	**Japan**	Department of Psychiatry, Jichi Medical University & Faculty of Sports Science, Waseda University	Randomized crossover	fNIRS	psychiatry	fatigue
Ohuchida et al.	2009	**Japan**	Department of Advanced Medical Initiatives, Faculty of Medical Sciences, Kyushu University	Non-experimental	fNIRS	surgery	psychomotor skill
Pelizzo et al.	2020	**Italy**	Department of Pediatric Surgery, ‘Vittore Buzzi’ Children’s Hospital, University of Milano	RCT	fMRI	surgery	psychomotor skill
Rotgans et al.	2019	**Netherlands**	Institute of Medical Education Research Rotterdam, Erasmus Medical Center, Rotterdam	Non-experimental	fNIRS	medical students –	clinical reasoning
Rourke et al.	2016	**Canada**	Department of Medicine, University of Alberta	Non-randomized controlled	EEG	cardiology and pulmonology	clinical reasoning
Shafiei et al.	2018	**USA**	Department of Urology, Applied Technology Laboratory for Advanced Surgery (ATLAS) Program at Roswell Park Cancer Institute	Non-experimental	EEG	surgery	psychomotor skill
Shetty et al.	2016	**UK**	Hamlyn Centre for Robotic Surgery, Imperial College London	Non-experimental	fNIRS	surgery	psychomotor skill development
Shewokis et al.	2017	**USA**	Nutrition Sciences Department, College of Nursing and Health Professions, School of Biomedical Engineering, Science and Health Systems, Drexel University	RCT	fNIRS	surgery	psychomotor skill
Turk et al.	2018	**USA**	VA Boston Healthcare System & Boston University School of Medicine	Non-experimental	EEG	medical students	declarative clinical knowledge
Veroude et al.	2013	**Netherlands**	Department of Educational Neuroscience, Faculty of Psychology and Education VU University Amsterdam	Non-experimental	fMRI	medical students	cognitive/emotional control
Wanzel et al.	2007	**Canada**	Department of Surgery, University of Toronto	Non-experimental	fMRI	surgery	visuospatial skill
Xu et al.	2018	**China**	Department of Military Psychology, Army Medical University, Chongqing, China.	RCT	EEG	medical students	well-being
Xu et al.	2019	**USA**	Center for Research and Innovation in Systems Safety, Vanderbilt University Medical Center	Non-experimental	fNIRS	anesthesiology	crisis event management team skills
Zhu et al.	2011	**Hong Kong**	Institute of Human Performance, The University of Hong Kong	Non-randomized controlled	EEG	surgery	psychomotor skill

EEG, electroencephalogram; fMRI, functional magnetic resonance imaging; fNIRS, functional near-infrared spectroscopy; NA, not applicable; OT, optical topography; RCT, randomized controlled trial.

* Other: Brain electric activity monitoring system designed by the researchers
